# *Cochliomyia hominivorax* aural myiasis in a 7-year-old traveler

**DOI:** 10.1016/j.idcr.2025.e02327

**Published:** 2025-07-20

**Authors:** Pranvera Feka-Homsy, Arnaud G. L’Huillier, Lionel Monod, Emile Monin, Nils Guinand, Jean-Marc Schwob

**Affiliations:** a Division of Otorhinolaryngology Head and Neck Surgery Institute, Clinical Neurosciences Department, Geneva University Hospitals and Faculty of Medicine, Switzerland; b Pediatric Infectious Diseases Unit, Department of Women, Child and Adolescent Medicine, Geneva University Hospitals and Faculty of Medicine, Geneva, Switzerland; c Department of Invertebrate Zoology, Natural History Museum, Geneva, Switzerland; d Division of Tropical and Humanitarian Medicine, Geneva University Hospitals, Switzerland; e Laboratory of Clinical Parasitology, Geneva University Hospitals, Switzerland; f Department of Clinical Sciences, Institute of Tropical Medicine, Antwerp, Belgium

**Keywords:** *Cochliomyia hominivorax*, Otomyiasis, Myiasis, Ear, Smith-Magenis syndrome, DNA-barcoding

## Abstract

• This is a very rare case of aural *Cochliomyia hominivorax* diagnosed in a pediatric-traveler outside endemic area.
• Unlike common travelers’ myiasis, *Cochliomyia hominivorax* can be invasive and destructive.
• Cytochrome-C oxidase-I (COI) DNA-barcoding may be a useful tool for diagnosing rare parasitosis.

## Case presentation

**Fig. 1:**
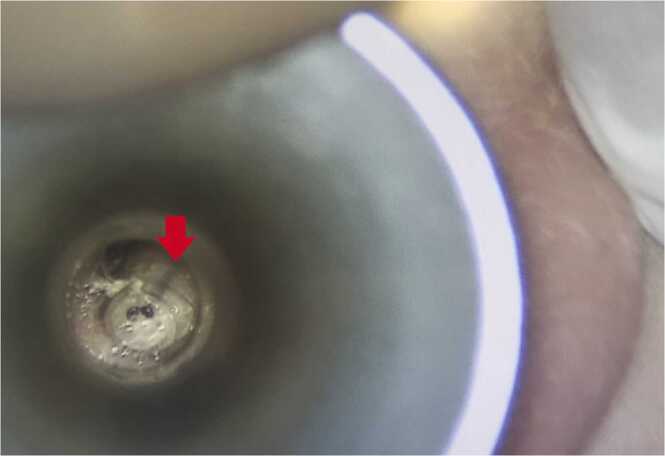
Otoscopic surgical view of the ear: the typical pigmented tracheal trunks (red arrow) of *Cochliomyia hominivorax* larvae can be seen following the anal plate.

**Fig. 2:**
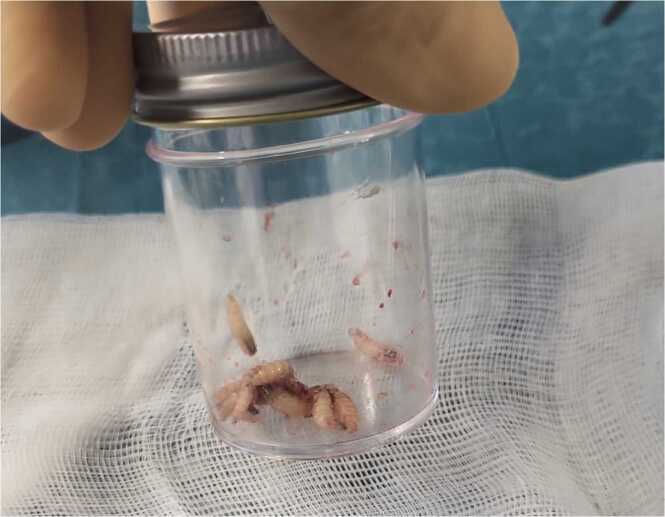
*Cochliomyia hominivorax* after extraction.

A 7-year-old female consulted at Geneva University Hospitals in Switzerland for acute right otorrhea three days after returning from the Brazilian Amazon region. The patient had Smith-Magenis syndrome, a complex neurodevelopmental disorder affecting multiple organs, and associated with a higher risk of chronic otitis media (COM). The patient had previously undergone bilateral middle ear surgeries with no documented recurrence. Under general anesthesia, otoscopy revealed 10 highly mobile larvae, each measuring around 15 mm within bloody-purulent secretions (Fig. 1 & Fig. 2). A small central perforation of the tympanic membrane was observed without an obvious sign of middle ear involvement.

Based on the clinical presentation, exposure, and morphology, American *Cochliomyia hominivorax* (New World Screwworm) and ubiquitous *Lucilia* spp. were suspected. As *C. hominivorax* was considered the most likely causal agent and posed the highest risk of tissue invasion, the patient underwent contrast-enhanced inner-ear/brain magnetic resonance imaging and CT-scan, which revealed no signs of deeper invasion. No additional surgical or pharmacological treatment was required, and the patient made full recovery. Definitive diagnosis was achieved using DNA-barcoding, a molecular technique for characterizing samples using sequences of the cytochrome-C oxidase-I (COI) gene in the mitochondrial genome [1] (Appendix 1).

## Discussion

Myiasis is a primarily zoonotic condition caused by larvae from several species from the insect order Diptera, feeding on live or necrotic tissues from the host [2]. Clinically myiasis is categorized as sanguinivorous, cutaneous (furuncular/migratory), wound, and cavitary and occur more frequently in tropical areas [2]. The burden of myiasis remains poorly documented in humans because of the stigmatization and low importance accorded to the disease [2].

*C. hominivorax* is an obligate wound myiasis affecting mostly bovine livestock but also other mammals, including humans [3]. Endemic to the New-World and now restricted to South America and the Caribbean, it is the first reported cause of myiasis in Argentina and Brazil [3]; Its diagnosis remains exceptional in the Old-World and in pediatric-travelers. In children, *C. hominivorax* has been described in association with malnutrition, autism-spectrum-disorders, or COM [4]; Additional risk-factors include suppurative/secretive wound [3]. Unlike common furuncular myiasis species such as *Dermatobia hominis*, these rapidly-growing larvae can cause deep and destructive lesions in healthy host tissue [2]. Infections involving the ear or nasal cavities may lead to bone destruction, and fatal central nervous system complications can occur [2], [3], [4], [5].

In this case, a new recurrence of COM was suspected to be the entry wound. Although suggestive morphological features were present (Fig. 1.), molecular biology allowed to ascertain the diagnosis. The short parasitic lifecycle (5–7 days from oviposition to pupation) makes *C. hominivorax* identification exceptional and challenging outside endemic areas.

## Authors contribution

PFH, NG, AL and JMS contributed to the patient care. LM performed the microscopic and molecular identification of the parasite. EM and JMS performed the literature search. JMS wrote the manuscript with the support of EM, LM, PFH and AL. PFH, NG and AL reviewed the manuscript. All authors contributed to the final version of the manuscript.

## Authors statement

Pranvera Feka-Homsy, Nils Guinand, Arnaud G. L’Huillier and Jean-Marc Schwob contributed to the patient care.

Lionel Monod performed the microscopic and molecular identification of the parasite. Emile Monin and Jean-Marc Schwob performed the literature search.

Jean-Marc Schwob wrote the manuscript with the support of Emile Monin, Lionel Monod, Pranvera Feka-Homsy and Arnaud G. L’Huillier.

Pranvera Feka-Homsy, Nils Guinand and Arnaud G. L’Huillier reviewed the manuscript.

All authors contributed to the final version of the manuscript.

## CRediT authorship contribution statement

**Pranvera Feka-Homsy:** Supervision, Investigation, Writing – review & editing. **Lionel Monod:** Formal analysis, Methodology, Investigation. **Arnaud G. L’Huillier:** Investigation, Writing – review & editing, Conceptualization, Supervision. **Emile Monin:** Investigation. **Jean-Marc Schwob:** Writing – original draft, Conceptualization, Supervision, Investigation. **Nils Guinand:** Writing – review & editing, Supervision.

## Consent to participate

Written informed consent has been obtained from the patient representatives.

## Declaration of author agreement

All authors have seen and approved the final version of the manuscript being submitted. They warrant that the article is the authors' original work, hasn't received prior publication and isn't under consideration for publication elsewhere.

## Sources of funding

This article received no specific grant from any funding agency in the public, commercial, or not-for-profit sectors.

## Declaration of Competing Interest

The authors declare that they have no known competing financial interests or personal relationships that could have appeared to influence the work reported in this paper.
