# Supplementary material for: Cochliomyia hominivorax aural myiasis in a 7-year-old traveler
Source: IDCases. 2025 Jul 20;41:e02327. doi: 10.1016/j.idcr.2025.e02327 (PMC12305604; doi:10.1016/j.idcr.2025.e02327)
Supplement: Supplementary file 1 — Supplementary material [file mmc1.docx]

**Appendix 1**

**DNA extraction, amplification, purification and sequencing.**

Total genomic DNA from one maggot was extracted with the E.Z.N.A. Tissue DNA Kit (Omega Biotek) following the manufacturer protocol. The two remaining specimens are stored in the collection of the MHNG (Museum d’Histoire Naturelle, Genève).

PCR amplifications of the mitochondrial cytochrome c oxidase I gene (COI) were carried out using the Taq PCR Core Kit (Qiagen) and the universal primer pair LCO1490/HCO2180 (Folmer et al., 1994).

**PRIMERS**

1) LCO 1490: 5’-GG TCA ACA AAT CAT AAA GAT ATT GG-3’

2) HCO2198: 5'-TA AAC TTC AGG GTG ACC AAA AAA TCA-3'

Cycles of amplification were carried out as follow: (1) 1 min at 98°C, (2) 10 s at 95°C (initial denaturing), (3) 30 s at 50°C (annealing), (4) 30 s to 1 min at 72°C (elongation) and (5) 10 min at 72°C (final elongation). Steps 2–4 were repeated 44 times.

Purification and sequencing of PCR products were performed at Macrogen Europe using the Sanger method (Sanger et al., 1977).

**Data post-sequencing sanitation and BLAST search**

Reverse and forward DNA sequences were aligned, manually edited, and a consensus of 701 bp (see below) was produced using Unipro Ugene v50.0 (Okonechnikov et al., 2012). A standard nucleotide BLAST search was then performed online on the NCBI database using the blastn algorithm (Zhang et al., 2000; Morgulis et al., 2008)

(<https://blast.ncbi.nlm.nih.gov/Blast.cgi?PROGRAM=blastn&PAGE_TYPE=BlastSearch&LINK_LOC=blasthome>). A total of 156 DNA sequences of *C. hominivorax* present in the database match with our own sequence. The first 100 show a 98% query cover and 98.98-99.56% base pair similarity, confirming the identification of the sample made by visual examination.

COI consensus sequence generated:

AAGATATTGGTACTCTATATTTCATTTTTGGAGCTTGATCAGGAATAATTGGTACTTCTTTAAGAATCTTAATTCGAGCAGAATTAGGACATCCAGGAGCATTAATTGGAGATGATCAAATCTATAACGTAATTGTTACAGCTCATGCTTTTATTATAATTTTCTTTATAGTAATGCCAATTATAATTGGAGGATTTGGAAATTGATTAGTTCCTCTAATACTAGGAGCTCCTGATATAGCTTTCCCACGAATAAATAATATAAGTTTTTGACTTTTACCTCCTGCATTAACTTTACTATTAGTAAGTAGTATAGTAGAAAATGGAGCTGGAACAGGATGAACTGTTTACCCTCCCCTATCTTCTAATATTGCTCACGGAGGAGCTTCTGTTGATTTAGCAATTTTTTCCTTACACCTAGCCGGAATCTCTTCAATTTTAGGAGCAGTAAATTTTATTACAACTGTAATTAATATACGATCAACAGGAATTACATTCGATCGAATACCTTTATTTGTTTGATCTGTAGTAATTACTGCTCTTTTACTTTTATTATCTTTACCAGTTTTAGCCGGAGCAATCACTATACTTTTAACAGACCGAAATTTAAATACTTCATTTTTTGACCCAGCAGGAGGAGGAGATCCAATCTTGTACCAACATTTATTTTGATTTTTTGGTCACCCCGAAAATTTAAAAAAA
